# Development and acceptability of behavioral interventions promoting mothers’ brushing of pre-school children’s teeth: The preparation phase of the multi-phase optimization strategy framework

**DOI:** 10.1186/s12903-023-03351-x

**Published:** 2023-08-31

**Authors:** Merna Ihab, Wafaa Essam El-Din Abdelaziz, Walid Hassan, Maha El Tantawi

**Affiliations:** 1 Department of Pediatric Dentistry and Dental Public Health, Faculty of Dentistry, Alexandria University, Alexandria University, Champollion St, Azarita, Alexandria Egypt; 2 Department of Psychiatry, Faculty of Medicine, Ain Shams University, Cairo, Egypt

**Keywords:** MOST, Oral hygiene, Early childhood caries, Acceptability, mHealth, Motivational interviewing

## Abstract

**Background:**

Early childhood caries can be prevented through regular parental-supported toothbrushing, indicating the importance of behavior modification interventions targeting parents. Mobile oral health (m-oral health) interventions are gaining increased popularity although their production is not always based on solid theoretical frameworks and evidence about the efficacy of individual intervention components is not available. The Multiphase Optimization Strategy (MOST) offers a framework to develop complex m-oral health interventions and assessing the efficacy of individual components.

**Aim:**

This study describes the development and assesses the acceptability of 3 intervention components using MOST to promote mothers’ brushing of their preschool children’s teeth.

**Methods:**

The Theory of Planned Behavior guided the development of 3 components: motivational interviewing (MI), storytelling videos (STVs), and oral health promotion messages (OHPMs). A researcher received training to conduct MI. Twenty-four OHPMs were developed, and 14 STVs scripts were developed based on the “And, But, Therefore” framework. A feasibility pilot study was conducted to determine the optimization objective and assess mothers’ preferences regarding the frequency and timing of receiving the intervention components. The mothers participated in a semi-structured interview to assess the acceptability of the components using 7 open-ended questions based on the framework of acceptability and thematic analysis was used to analyze the qualitative data. The mothers also responded to questions assessing the perceived and experienced acceptability of the components using close-ended questions. Descriptive statistics were presented as means and standard deviations for continuous variables and median and interquartile range for categorical variables.

**Results:**

Sixteen mothers were included. The mothers expressed positive affective attitude towards the interventions. They felt the components served as “good reminders” to brush their children’s teeth. However, “time” was a burden for the mothers. 80% of the mothers preferred receiving the OHPMs and STVs once per week, from 8 pm to 2 am (50%), and 60% indicated they can set 15–30 min to receiving the interventions.

**Conclusion:**

The 3 components were acceptable to the mothers. The OHPMs and STVs will be sent to the mothers once per week, between 8 pm to 2 am. The MI and follow-up phone calls will be limited to 15 min.

**Supplementary Information:**

The online version contains supplementary material available at 10.1186/s12903-023-03351-x.

## Background

Early childhood caries (ECC) is the presence of a primary tooth that is carious (non-cavitated or cavitated), missing due to caries, or filled in a child under the age of six years [1]. ECC can be prevented by behavior change interventions targeting regular tooth brushing using fluoridated toothpaste [2]. However, children are dependent on their parents, and through primary socialization, learn the norms, beliefs, and health behaviors of their families [3]. Thus, it is important to develop interventions targeting parents to adopt positive oral heath behaviours for their children to prevent ECC.

Printed materials such as posters, flyers, leaflets, and mass media have been traditionally used for oral health promotion [4]. Also, motivational interviewing (MI) can change oral health knowledge, attitudes, and behaviors of parents of preschool children [5], thus, preventing ECC [6].

Dental health education messages delivered via mobile phones are useful with parents of young children [7], improving knowledge of children’s oral health [8]. Text messaging has multiple attractive features. They require less resources than interventions based on in-person delivery, can be automated for delivery to targeted populations at pre-specified times, reach a broad audience at reduced cost and their frequencies can be tailored according to the complexity of targeted behaviors and interventions. They can also be sent through social media platforms and serve as reminders for behaviours.

Video consumption is rapidly growing and online videos constitute over 75% of all global internet traffic [9]. Storytelling in videos can positively impact patients’ education and influence behavior, providing a simulation to real life that immerses viewers in experiences, exposes them to new places and situations, and makes viewers care more about the issue [9]. Videos were also effective in improving oral health knowledge [10].

With the increased use of mobile phones, mobile health (mHealth) applications were developed. mHealth includes medical and public health practices supported by mobile devices, such as mobile phones, personal digital assistants, wireless devices, and patient monitoring devices [11]. However, mHealth products, including videos and messages, are rapidly evolving at the expense of being based on sound theoretical frameworks or tailored to participants’ needs [12]. Solid theoretical frameworks can support the production of mHealth interventions but are often overlooked and seldom reported [13].

It is estimated that 67% of Egyptian preschool children suffer from untreated ECC [14]. In addition, Egyptian school children aged 11–15 years old rank the second lowest worldwide in the percentage of children with regular toothbrushing (32.1%) [15]. The large number of Egyptian preschool children affected by ECC, and the prevalent suboptimal level of oral hygiene call for interventions to affect behavior change and instill proper oral hygiene practices. In addition, the high level of internet penetration in Egypt [16] makes it possible to use m-oral health applications to promote oral health.

The Multi-Phase Optimization Strategy (MOST) is an engineering framework to develop and assess the efficacy of separate components of an intervention package. The MOST framework consists of three phases: *preparation, optimization, and evaluation*; in which an intervention is developed then optimized in a subsequent optimization factorial trial before it can be tested as a package in a randomized clinical trial (RCT) in the evaluation phase [17]. An optimization objective is also specified in the preparation phase to ensure that the resulting package is affordable, scalable, and efficient. The preparation phase in this study includes: (1) developing a conceptual framework to guide the design of the intervention components, (2) developing the intervention components, (3) conducting a feasibility pilot study to determine the acceptability of the intervention components and the preferred frequency and timing of receiving the intervention components and (4) setting an optimization objective [18].

In this preparation phase study, two m-oral health components (oral health promotion messages (OHPMs) and STVs) and MI sessions were developed and their acceptability to mothers of preschool children was assessed. The current study is the first to apply the MOST framework in the field of dentistry and will be followed later by a subsequent optimization factorial trial.

## Methods

This paper was written in accordance with the MOST PREParation REPorting checklist [19] (Appendix 1) and the methods outlined in our previously published protocol [18] .

### Step 1: developing a conceptual framework

**Fig. 1:**
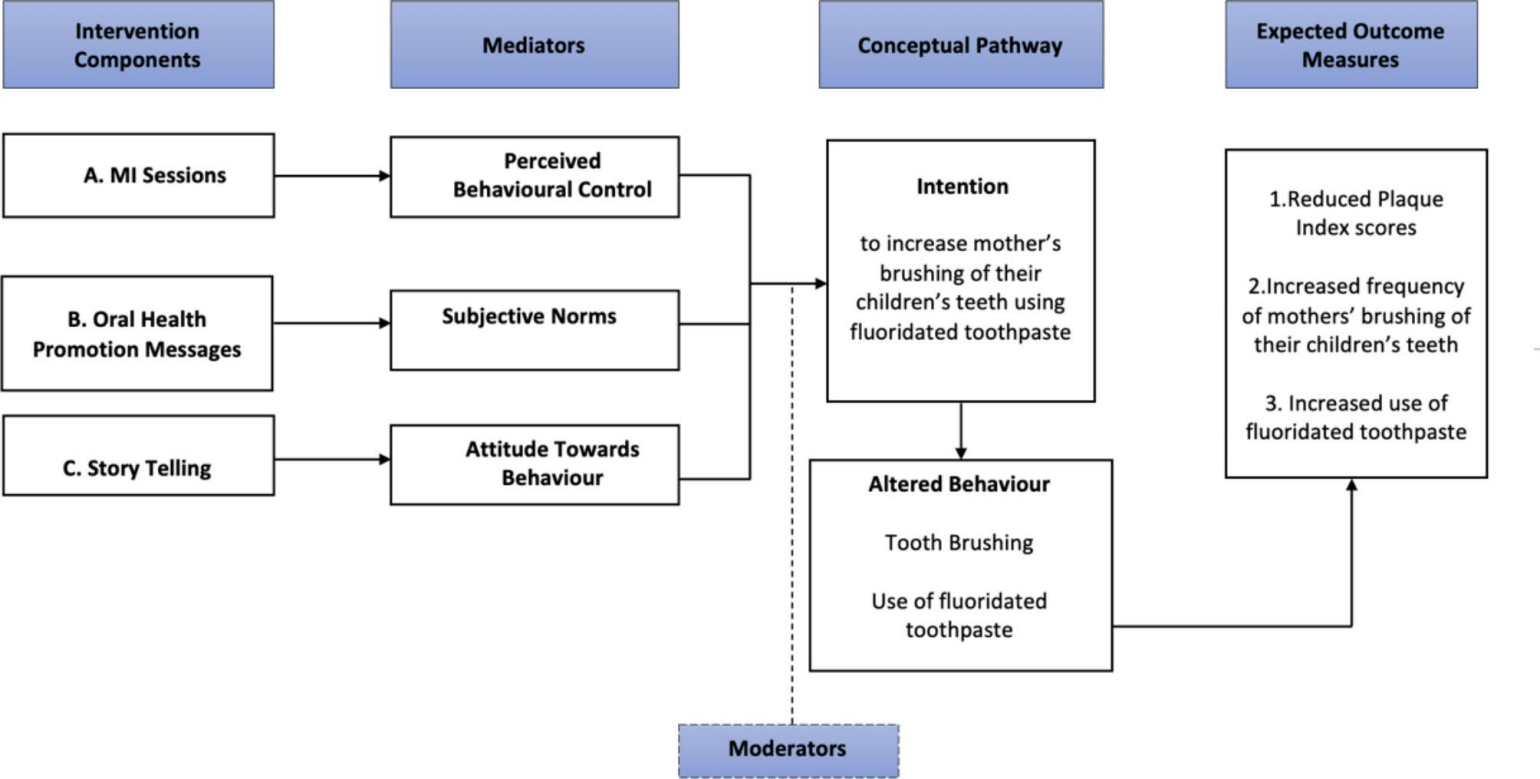
Conceptual framework guiding the design of the intervention components

The conceptual framework guiding the development of the intervention components was grounded within the Theory of Planned Behaviour (TPB) [20]. The TPB indicates that intentions are precursors of behaviours, making intention a valid indicator of the impact of an intervention [21]. Intentions are affected by perceived control on intended behaviour, attitude towards behaviour, and subjective norms [20]. We proposed three intervention components targeting each construct of the TPB: MI targeting perceived control, OHPMs targeting subjective norms, and storytelling videos (STVs) targeting attitude (Fig. 1).

The proposed intervention components were selected based on evidence from previous studies. A systematic review [22] highlighted that MI outperformed traditional oral health education in improving patient behaviors and oral health perceptions and enhancing clinical indicators such as plaque index, gingival index, and bleeding on probing. A study [23] proposed that messages grounded in subjective norms were the most effective in inducing behaviour changes. The STVs were based on a study using the TPB to develop short-format scientific videos [9]. The respective intervention components were then aligned with the constructs of the TPB by checking the validity of each component as will be detailed in the following paragraphs.

### Step 2: developing intervention components

#### Motivational interviewing

The first author had training [24] on how to provide MI and was further trained by a certified MI expert (WH). Potential scenarios of encounters with mothers were discussed in five meetings before the study. Four face-to-face MI sessions with mothers were audio-recorded after obtaining written consent to assess the MI sessions’ fidelity. The expert listened to the audiotapes and reviewed the researcher’s technique, ensuring that MI core skills (asking, listening, affirming, and summarizing) were used. In the fully powered optimization factorial trial, one in-person MI session will be offered to mothers to establish rapport with mothers, encourage them to talk about their oral health habits and discuss options and strategies to improve their children’s oral health with the ultimate goal of developing mothers’ perceived control over brushing their children’s teeth. After this first in-person session, the mothers will receive follow-up phone calls every 2 months for 6 months to reinforce commitment to the new behaviour (Table 1). The duration of the MI session was determined in this feasibility pilot study.

#### Oral health promotion messages

Twenty-four unique OHPMs were developed to promote mothers’ brushing of their children’s teeth based on the perception of subjective norms. The messages were based on previous studies [25, 26], and were modified to fit the current study. The messages were formulated in Egyptian Arabic and without jargon to increase participants’ interest, acceptance and understanding. The messages were phrased along variations in three areas: (1) content, discussing the impact of oral health on child’s self-confidence, facial aesthetics and speech, and the cost of treating ECC, (2) the group within which norms were framed, including family, friends, or the community in general, and (3) sentiment, whether positive showing the benefits of good oral health or negative warning against the outcomes of poor oral health. The following is an example of an OHPMs with positive sentiment showing the impact of facial aesthetics due to good oral health and how they are perceived by friends: *“Brushing your children’s teeth twice a day with a toothbrush and toothpaste help make their smile more beautiful in front of their friends”.* The content validity of the OHPMs was assessed by seven postgraduate students of Paediatric Dentistry (3) and Dental Public Health (DPH) (4). The mean content validity index (CVI) of all OHPMs was 0.878. OHPMs with low CVI (< 0.83) [27] were modified or removed. The modified list of OHPMs was sent again to the same evaluators for re-assessment. The final CVI was 0.98, which was acceptable [27]. The final list of OHPMs is available in Appendix 2.

In the optimization factorial trial, each mother allocated to receive the OHPMs will be exposed to all 24 OHPMs sent via WhatsApp messenger [28]. The frequency and timing of receiving the OHPMs was determined in the feasibility pilot study (Table 1).

#### Storytelling videos

The ABT (and, but, therefore) model of storytelling [29] was used to develop 14 scripts narrating the experience of mothers whose children have ECC, e,g., *“Plaque causes ECC AND the child suffers BUT brushing can remove plaque and prevent caries THEREFORE it is important for mothers to brush their children’s teeth twice daily.”* Those scripts were used to develop videos to induce behaviour change by applying the SUCCESS criteria [30]. SUCCESS is an acronym for: **S**imple, includes **U**nexpected information, **C**redible, has a **C**oncrete narrative, **E**motional, based on **S**cientific evidence, and tells a **S**tory. **S**imple language was used for mothers with average (middle/ high school) education. The videos included **U**nexpected narratives intended to push mothers to improve their children’s oral hygiene, e.g., a child subjected to bullying by his peers due to the appearance of his carious teeth and how this had a negative psychological impact on the child. **C**redibility was promoted by featuring a dentist in his clinical attire talking in his dental clinic. We used a **C**oncrete narrative about, for example, how sugars release acids that remove tooth material. An **E**motional feature was added by showing happy little children with good oral health or mothers talking about how they suffered because of ECC. **S**cientific content was based on facts derived from studies and all this was included in a **S**tory, like one mother would tell another while waiting for her turn at the clinic. The scripts were developed by a panel of six DPH specialists and assessed for content validity by experts in Paediatric Dentistry and DPH ranging in number from 4 to 9 per video with good content validity (mean CVI = 0.98) [27].

The mothers were videotaped while responding to the questions of a structured interview. However, most mothers replied in short, yes/ no answers that did not produce narratives to frame the stories in the scripts. Because no budget was available to hire professional actors, the scripts were given to a number of DPH postgraduate students who rehearsed and were videotaped in their roles as mothers. A professional video editor edited the raw footage of the scripts, trimmed the videos, cut out pauses, and added the University logo and background music. This produced 24 short (1–2 min) videos, with about 2 videos showing the story of each script, in addition to a video introducing the series of STVs to audience. The links to the videos are in Appendix 3.

The mothers allocated to receive the STVs in the optimization factorial trial will receive the 24 videos via WhatsApp messenger. The frequency and timing of receiving the STVs was determined in the feasibility pilot study (Table 1).

**Table 1:** Summary of developed intervention components for optimization trial

Intervention component	Target	Frequency and timing of receiving the intervention	Mode of Delivery
MI	Enhance mother’s perceived control of brushing her child’s teeth	1 face to face session + 3 phone calls	In person + phone calls
OHPMs	Reinforce mother’s perception that brushing child’s teeth is considered important to significant others	*determined in the feasibility pilot study*	Electronic messages via WhatsApp messenger platform
STVs	Change the attitude of mother’s towards brushing her child’s teeth	*determined in the feasibility pilot study*	Electronic messages via WhatsApp messenger platform

### Step 3: the feasibility pilot study

#### Participants

Mothers visiting the clinic of the Department of Paediatric Dentistry and Dental Public Health, Faculty of Dentistry, Alexandria University, were conveniently selected and invited to participate in the feasibility pilot study. Recruitment stopped when information saturation occurred after including 16 mothers [31]. Mothers were eligible if they had children aged 2–5 years, if they were literate, owned a smart phone with WhatsApp messenger application or where WhatsApp messenger could be installed. Mothers were excluded if their children were physically disabled, medically compromised, or needed an emergency dental treatment. Prior to the commencement of the study, the Research Ethics Committee, Faculty of Dentistry, Alexandria University approved the study (#0273-07/21). The mothers signed an informed consent before joining the study.

**Fig. 2:**
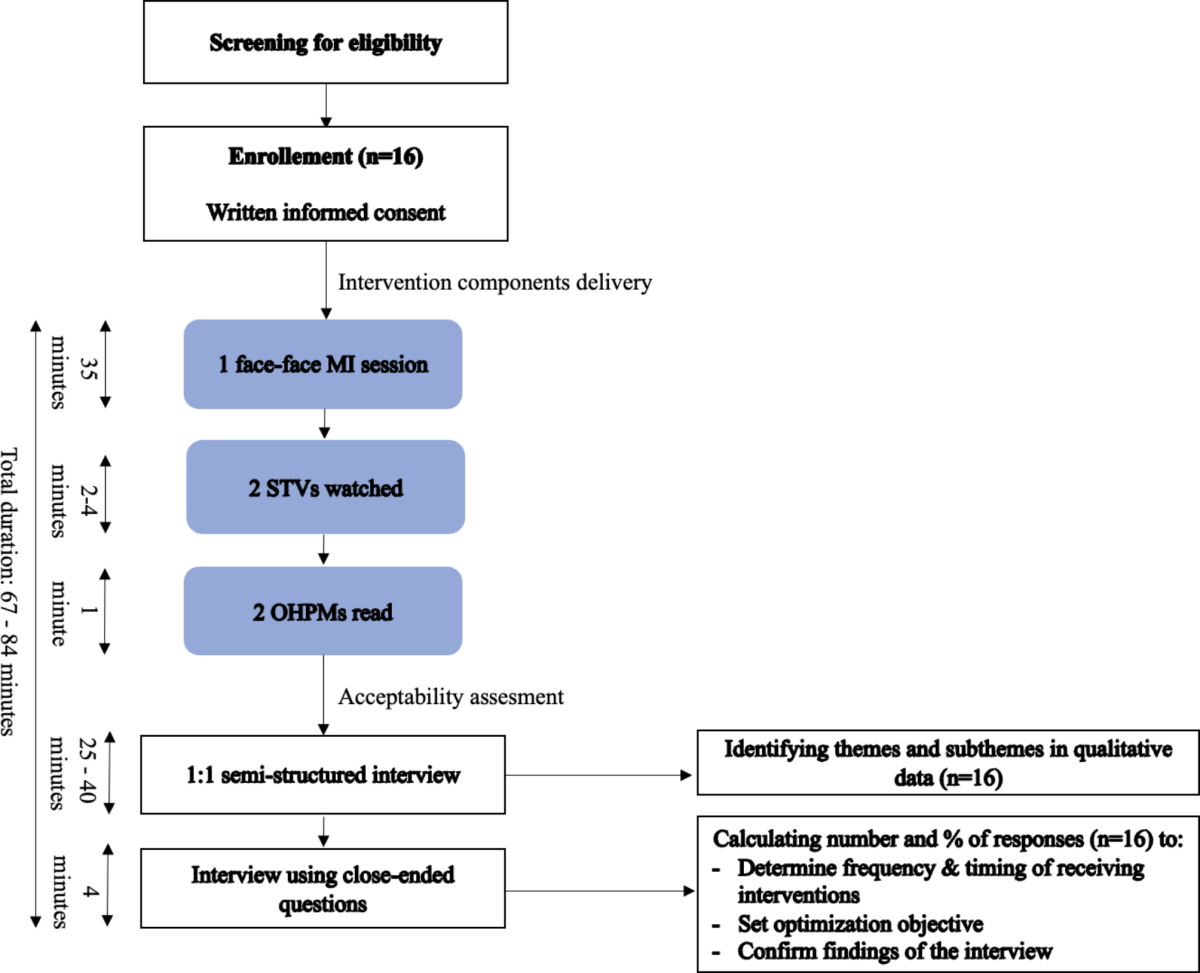
The stages of the feasibility pilot study

### Interview procedure

Figure 2 illustrates the stages of the feasibility pilot study. Each mother received two randomly selected OHPMs, two randomly selected STVs, and a 35 min MI session. Afterwards, the mother participated in an audio-recorded, 1:1, 25–40 min long, semi-structured, face to face interview conducted by a trained female researcher (HH) with master degree in DPH who was familiar with the study objectives and methods. The interviews were conducted in a quiet place beside the clinic waiting area. The first author took field notes during the interviews and audio-recorded them after obtaining the mothers’ consent. Seven open-ended, previously tested, questions were used to assess mother’s acceptability of the intervention components. The questions were based on the framework of acceptability (TFA), developed by Sekhon and colleagues [32] and included questions assessing mothers’ affective attitude, burden, perceived effectiveness, ethicality, intervention coherence, and opportunity costs (Appendix 4). The mothers also responded to a paper-based questionnaire (Appendix 5). The same previously tested, open-ended questions based on the TFA from the interviews were used as close-ended questions, to assess mother’s perceived and experienced acceptance of the intervention components. The responses were based on a 5-point Likert scale (completely agree to completely disagree). The questionnaire was validated by a panel of DPH specialists before the feasibility study and was shown to have good validity (CVI = 0.93) [27]. After the mother participated in the semi-structured interview, the same researcher (HH) presented the same questions to the mothers along with available responses. The mothers selected one response option, which was then recorded in the paper-based form by the researcher. Later, the responses were entered into an excel sheet. The inclusion of the questionnaire was to further aid interpretation and triangulation. The aim was not to collect new information but to confirm information already collected in the interview.

### Step 4: setting an optimization objective

The components were designed to be offered as part of a preventive package for mothers of young children in the Mother and Child Health centres of the Ministry of Health and Population in Egypt. The dentists employed in these facilities are paid salaries that are neither linked to the number of patients they manage, the type of services they offer nor the number of procedures they perform. Thus, the critical factor determining the uptake of the intervention components would depend on the mothers’ willingness to engage with the components. Time was the most important factor for this engagement because mothers are usually busy during the visit to the centres and try to complete all required steps (registration, immunization, weight and height measurements and general physical examination of the children) in the shortest time. The questionnaire that was used to assess perceived and experienced acceptability of the intervention components (Appendix 5) also assessed the preferred frequency and timing of receiving the intervention components, and the total time a mother is willing to spend receiving the components to set the optimization objective.

### Data analysis

The audio recordings were transcribed verbatim in Arabic to minimize the loss of meaning and depth of responses and analysed inductively at a semantic level [33, 34]. Two researchers (first and last authors) familiarized themselves with the data independently, identified initial codes and combined the codes to form common recurring themes and patterns. Key quotes were translated to English for reporting. For quantitative analysis, descriptive statistics were presented as frequencies, percentages, median, and interquartile ranges. SPSS MacBook (version 25) [35] was used for analysis.

## Results

Most (68.8%) mothers were between 25 and 34 years of age, married (93.8%), with college/ university degree or higher (50.0%) and housewives (62.5%, Table 2).

**Table 2:** Demographic characteristics of the participating mothers (n = 16)

Characteristics	n (%)
Age groups	
25–34 years	11 (68.8)
35–44 years	5 (31.2)
**Marital status**	
Married	15 (93.8)
Divorced	1 (6.2)
**Number of children: Mean (SD)**	2.2 (1.0)
**Father’s Education**	
High school	7 (43.7)
College/University and higher	9 (56.3)
**Mother’s Education**	
Middle school	3 (18.8)
High school	5 (31.2)
College/University and higher	8 (50.0)
**Mother’s Job**	
Housewife	10 (62.5)
Works outside home	6 (37.5)

We identified two main themes with underlying sub-themes related to how mothers perceived the intervention components:

### Theme 1. Features of the components related to perceived impact

#### Theme 1.1 support for mothers to brush their children’s teeth

The mothers had positive perception of the components, expressing that they were satisfied with them, and that the interventions motivated them to brush their children’s teeth *“When you send me something occasionally, and I see it, it makes a difference and encourages me to brush their teeth (participant 2)”*, and acted as reminders *“It is something that makes me remember and reminds me when I forget (participant 1)*”. The interventions also provided confirmation supporting them to take care of their children’s health. *“I feel like I know this information, but I feel you gave me a push to take care of their teeth and honestly, I am happy (participant 6).”* Some mothers emphasized the importance of face-to-face MI sessions for motivation, *“the interview is very nice (participant 5)”, “the interview is the best (participant 7).”*

### Theme 1.2 explaining harmful effects of caries had great impact

The participants emphasized that interventions explaining how the lack of toothbrushing negatively impacted a child’s health are convincing. The mothers stated that understanding the potential harm resulting from poor oral health had a stronger motivational effect compared to simply hearing a story. *“The harm, if it is more, is more motivating than having one tell a story, because the harm is what makes mothers more motivated”*. The mothers also highlighted the importance of focusing on the negative consequences of caries on various aspects of their child’s life. *“The impact on eating, school, and concentration because of cavities, …. [should be highlighted] … because school is very important (participant 1)”.*

### Theme 1.3 relatable and real storytelling videos

The mothers commented on how the STVs were relatable and similar to real life experiences. *“My son Mohamed, for example, is exactly like the boy in the video (participant 7)”*. The mothers felt that the stories depicted in the videos were authentic and genuine, and some shared personal experiences. One mother recounted an experience she had with a child who suffered from stuttering. She felt that witnessing such tangible stories would convince many mothers to take immediate action and take care of their children’s teeth. “*By the way, the story of the stuttering that I saw in this video… I had a personal experience with a child who was the same way, and his front teeth were all decayed like that, and the way he spoke, even though he was somewhat older…… It was very clear to us that he was embarrassed. This is tangible and is something that would make any mother take care of her child’s teeth immediately (participant 8).”*

### Theme 1.4 advantages of electronic interventions

The mothers explained that mHealth interventions were easily accessible and no effort would be exerted to participate in the interventions using them. *“I hold my mobile phone all day, so there is no effort (participant 13)”*. The mothers highlighted that almost all people nowadays have internet connection at home, and there was no additional cost associated with the mHealth interventions. *“There is no [additional] cost. We hold the mobile phone in our hands all day long. Instead of playing games, it is better to see something useful for a change (participant 4)”.* Participants also felt the importance and usefulness of sharing dental health information on social media platforms, since technology is the main method of communication between people nowadays, regardless of their social and educational level. *“I want you to upload a video with the information you just told me (participant 8)”, “I wish you would upload the videos on the internet so that mothers could learn how to deal with their daughters and brush their teeth. Encourage the mothers; there are mothers who do not know [how to take care of their children’s teeth] (participant 9)”.*

### Theme 2. Time and commuting as problems with the intervention components

Time and commuting were the main problems against getting involved with the intervention components, especially face-to-face MI sessions and MI follow-up calls. *“It is very difficult for me to come here; I have to wake up early, and so does the child (participant 6).”* Others tried to find a solution, *“[Coming to the] interviews is a bit of a problem, but this may be solved when the vacation comes (participant 1),” and “I have two days off per month and can use these to come here to gain experience (participant 4).”* Working mothers felt that the only free time they have was at night, when they had no work and their spouses were not home. Others felt that their free time was “precious” and “sacred” and spending it answering follow-up MI phone calls would be a commitment and a burden, unlike viewing videos and messages on their mobile phones whenever they were free. On the other hand, some stay-at-home moms felt they could allocate some free time to receiving the interventions *“I am a housewife and I have a lot of free time, so I don’t feel like it troubles me (participant 5)”, “I am a housewife, and I do not have meetings, nor do I have interviews with someone, only my home, my husband, and my children. My connections and trips are very few. I feel that I can devote time to the things I hear or read (participant 8).”*

The mothers’ responses to the acceptability questionnaire are tabulated in Table 3. The analysis showed high median scores for *affective attitude, intervention coherence and perceived effectiveness*, indicating high acceptability of the three components. *Perceived burden*, *ethicality, and opportunity cost* had low median scores, indicating minor perceived problems in these areas with the three components.

**Table 3:** Mother’s acceptance of the intervention components

Acceptability Construct	Items	Median (IQR)		
		MI	OHPMs	STVs
Affective attitude	1. Enjoyed receiving it	5 (0.0)	4 (1.0)	5 (1.0)
Perceived burden	2. Exerted effort to receive it	1(1.7)	1 (1.0)	1 (1.0)
Intervention Coherence	3. Easy to understand and follow content	5 (0.7)	5 (1.0)	5 (0.0)
Ethicality	4. Inappropriate for my values ​​and the way I live	1(0.0)	1 (0.2)	1 (0.0)
Opportunity Cost	5. Gave up important things ​​to receive it	1(0.0)	1(0.0)	1 (0.0)
Perceived effectiveness	6. Acts as motivation for other mothers	5 (0.0)	5 (1.0)	5 (0.0)

Most mothers preferred receiving the m-oral health components once per week (80.0%). Half the mothers preferred receiving the interventions from 8pm to 2am emphasizing that they have busy schedules, and late evenings provided them with an opportunity to check their phones and engage better with the messages and videos sent.

### Setting the optimization objective

60.0% of the mothers indicated that 15–30 min is the maximum time they were willing to spend receiving the components (Table 4). Therefore, the optimization objective was set to be the lowest plaque index score that can be obtained provided that the mother does not spend more than 15 min receiving the intervention components.

**Table 4:** Optimization Objective

		N (%)
**Preferred maximum frequency of receiving the videos and messages**	Once per week	12 (80.0%)
	3 times per month	2 (13.3%)
	2 times per month	1 (6.7%)
**Preferred time of receiving the videos and messages**	8am to 2pm	3 (21.4%)
	2pm to 8pm	3 (21.4%)
	8pm to 2am	7 (50.0%)
	2am to 8am	1 (7.2%)
**Maximum total time to spend receiving intervention components**	> 1.5–2.5	5 (33.3%)
	> 15–30	9 (60.0%)
	> 30–45	1 (6.7%)

Numbers do not add up to the total of 16 mothers because of item non response

## Discussion

### Summary of findings

This study is the first to use the MOST framework with a step-by-step guide to develop m-oral health components based on a theoretical framework and responding to patients’ preferences. Our study follows the recommendations for developing and designing behaviour change interventions [36], and serves as a model for the development of future m-oral health promotion interventions [37]. In this phase of MOST, we have successfully developed 3 valid components with good acceptability to mothers, and valid tools to assess them, in addition to setting the optimization objective.

The developed intervention components target mothers who are seeking extensive dental treatment for their children (i.e., extractions and pulpotomies) who were visiting the pediatric dentistry clinic in the college and might, therefore, be motivated to treat their child’s teeth. However, they do not adequately brush their children’s teeth and may not necessarily be seeking preventive care in this clinic. The literature shows that Egyptians are mainly symptom- driven and postpone dental visits until they experience pain [38]. Thus, the mothers in the study setting would benefit from behavior modification interventions that motivate them to take care of their child’s teeth. In addition, the developed m-oral health components can be added to the portfolio of existing m-oral health resources for the oral health research community targeting Arabic speaking populations.

The STVs developed in this study targeted the attitude construct of the TPB. The STVs scripts were based on the ABT model of storytelling and were validated whereas the SUCCESS criteria were used to develop the videos. Our study fills a knowledge gap by providing guidance on the development of STVs to promote oral health and modify oral health behaviors. After the recent focus on m-oral health applications [38], more videos are expected to be developed to target oral health promotion and our resources may be of help in this field. Before the present study, the existing literature focused on developing educational videos to disseminate oral health knowledge in hospital setting [10]. We also identified a study using social stories [25] to explain adequate toothbrushing frequency and demonstrate correct toothbrushing techniques for children. To our knowledge, no studies used videos to tell a story to promote healthy behaviors, especially for mothers of preschool children.

In the present study, we developed another m-oral health component: OHPMs to promote toothbrushing by targeting the impact that perceived norms would have on mothers in addition to assessing the validity of the developed OHPMs. In collectivist societies such as the Egyptian society, prevailing norms may play an important role in shaping people’s health behaviors. The mothers may be affected by OHPMs linking oral health impact on life to norms and opinions of people in the mother’s circle. The OHPMs developed in the present study were based on perceived norms as a single construct of the TPB. A previous study [23], assessed the perceived effectiveness of a series of theoretically driven messages based on all three constructs of the TPB, targeting young adult’s routine dental checkups. Also, and a recent systematic review [39] reported the use of digital and virtual technologies, such as apps, text messaging, videos, and computer-aided learning, to prevent and promote oral health among adolescents and young adults using orthodontic appliances, where messages served as oral hygiene reminders/ reinforcements. However, the derivation of message content in previous studies is often not clearly reported or totally absent, leaving it unclear how and why the messages were developed and implemented [40]. Our OHPMs, therefore, provide resources for oral health promotion among Arabic speaking mothers and are particularly useful considering the large number of young children in Arabic speaking countries.

The MI sessions targeted the behavioral control construct of the TPB and were checked for fidelity. In a recent study [41], the fidelity of MI sessions with caregivers of young children and MI sessions by experts versus beginners were associated with greater reduction in sugary foods consumption although the difference was not significant. Also, MI had a significant effect on preventing ECC that depended on the quality of MI [42]. The effect of MI on improving oral health outcomes may be conflicting partly because of the unclear strategies of assessing the fidelity of MI-based interventions [43]. It is recommended to ensure that future MI-interventions are based on a solid theoretical framework and reporting.

The findings of our feasibility pilot study show that the intervention components were well accepted by mothers. Time was the only issue identified by mothers that may negatively impact their engagement with the components, which supports our selection of time as an optimization objective. Evidence showed that there is no consensus in the literature on the most appropriate timing, frequency, and nature of message delivery [39]. Although it is important to make sure that oral health promotion interventions are relevant to participants’ preferences and needs, a recent systematic review showed that most m-oral health and teledentistry interventions were too generalized [39]. To overcome the time barrier identified in the study and build on information gained while setting the optimization objective, we plan to send the STVs and OHPMs to mothers’ mobile phones according to their preferred schedule and free time. The in-person and follow-up MI sessions will also be limited to 15 min to reduce the time mothers spend on the intervention components, especially working mothers.

### Strengths and limitations

The main limitation of the study is that the intervention components were assessed only for validity and acceptability in a feasibility pilot study that was not powered to assess efficacy. Efficacy assessment is the aim of the next phase of MOST which is the optimization factorial trial. The findings related to acceptability and the optimization objective are expected to be applicable to target populations of similar background and further evidence is needed about the applicability among Arabic speaking participants in different settings and countries. Despite these limitations, the study has several strengths. It presents the first attempt to develop a complex behavior modification intervention using the MOST framework adopting a rigorous methodology for intervention optimization. As part of the MOST framework, we developed three intervention components that were theoretically grounded, based on participants’ preferences, and feasible to deliver with fidelity in an optimization factorial trial. The acceptability of these components may increase the likelihood that mothers engage with the intervention to improve and maintain the target behaviour. The employment of the MOST framework, thus, holds considerable promise in the development of future high-quality optimized oral health behaviour modification interventions that are grounded within solid theoretical frameworks, effective, and readily implementable in their intended contexts.

## Conclusion

The present study showed that the MOST framework could be used to develop multiple components of a behaviour modification intervention: MI, OHPMs, and STVs, to enhance mothers’ brushing of their preschool children’s teeth. The feasibility pilot study demonstrated the validity and acceptability of the developed intervention components. The greatest proportion of mothers preferred to receive the intervention components once per week between 8 pm to 2 am. The optimization objective was set as the best expected oral health outcome that can be achieved provided that the mothers do not spend more than 15 min receiving the intervention.

### Electronic supplementary material

Below is the link to the electronic supplementary material.

Supplementary Material 1

## Data Availability

All data generated or analysed from this study are included in this published article.

## Abbreviations

***WHO***: World Health Organization
***ECC***: Early Childhood Caries
***mHealth***: Mobile health
***OHPMs***: Oral health promotion messages
***MOST***: Multi-Phase Optimization Strategy
***TPB***: Theory of Planned Behaviour
***MI***: Motivational Interviewing
***STVs***: Storytelling videos
***CVI***: Content validity index
***ABT***: And, But, Therefore model
***TFA***: The Framework of Acceptability
