# Supplementary material for: Development and acceptability of behavioral interventions promoting mothers’ brushing of pre-school children’s teeth: The preparation phase of the multi-phase optimization strategy framework
Source: BMC Oral Health. 2023 Aug 31;23:616. doi: 10.1186/s12903-023-03351-x (PMC10470132; doi:10.1186/s12903-023-03351-x)
Supplement: Supplementary file 1 — Supplementary Material 1 [file 12903_2023_3351_MOESM1_ESM.docx]

**Appendix 1**

**PREP REP checklist**

|  | **Item #** | **Recommendations** | **Lines** |
| --- | --- | --- | --- |
| **Introduce the MOST framework** | 1 | 1. Identify the use of the multiphase optimization strategy (MOST) with appropriate citation (s) 2. Label aspects of current or prior work that the research team considers preparation for an optimization phase as the preparation phase | 96 - 100    107 - 109 |
| **Describe the research series** | 2 | 1. Provide context for the current work in the introduction or background and clearly label prior, ongoing, or planned work as preparation where appropriate 2. Describe the trajectory of the research program leading up to the current work. 3. provide citations for any prior or associated work whether it was completed by the research team or by the others (e.g., literature reviews, formative research, pilot studies, trials) 4. Connect the trajectory of the research program to the MOST phase(s) by labelling prior and current work with the appropriate phase (e.g., preparation phase, optimization phase) including any iterative movement between phases 5. if space is available, provide example(s) of how prior work was translated into elements of the conceptual model and/ or the optimization objective | 107 - 109  113  96 - 100  115-130,  Figure 1 |
| **Clearly define the conceptual model** | 3 | 1. Use the term conceptual model to identify the theory or framework guiding the selection of the components, mediators, and outcomes for optimization. 2. Provide a detailed description of the conceptual model including a figure or diagram 3. Identify and describe the intervention components and levels 4. Identify primary (and secondary) outcomes being targeted by the intervention components including description of assessment tools 5. Describe how each intervention component is purported to affect each outcome including descriptions of how relevant mediators will be assessed | 115-130,  Figure 1  115-130,  Figure 1 |
| **Optimization Objective** | 4 | 1. Use the term optimization objective to identify the factors that will guide the selection of intervention components after the optimization trial 2. Provide an operational definition of the optimization objective   Include specific criteria, if known, for determining which components will be retained in optimized intervention such as benchmarks related to effectiveness, scalability, and/or efficiency.   1. Describe the rationale for the selection of the optimization objective | 246-259  354-356  246-259 |

**Appendix 2**

**Validated OHPMs**

| English Message | Arabic Message | Target | Target | +/- |
| --- | --- | --- | --- | --- |
| 1. Brushing your children’s teeth twice a day using a toothbrush and toothpaste will help make their smile more beautiful in front of their friends and neighbours. | غسيل أسنان طفلك مرتين يومياً بالفرشاة ومعجون الأسنان هيساعده ان ابتسامته تكون أجمل أمام أصدقائه وجيرانه | aesthetics | friends | positive |
| 1. Your friends and neighbours pay attention to the appearance and cleanliness of your child's teeth. Give your children a better look by cleaning their teeth twice everyday using a toothbrush and toothpaste. | أصدقائك وجيرانك بينتبهوا لمظهر أسنان طفلك ونظافتها. اعطيه مظهر أفضل بغسيلها بانتظام كل يوم مرتين بالفرشاة ومعجون الأسنان | aesthetics | friends | positive |
| 1. Healthy and clean teeth will help your children blend with their neighbours and friends and feel confident in themselves. That's why it’s important to help your children brush their teeth twice a day using a toothbrush and toothpaste. | الأسنان السليمة والنظيفة هتساعد طفلك يندمج مع جيرانه وأصحابه ويشعر بثقة في نفسه. عشان كدة ساعديه إنه يغسل أسنانه مرتين كل يوم بالفرشاة ومعجون الأسنان | confidence | friends | positive |
| 1. Protecting your children’s teeth from decay, ensuring it’s cleanliness, and brushing it twice a day using a toothbrush and toothpaste, will allow them to play with their friends and neighbours confidently, without feeling embarrassed. | حفاظك على أسنان طفلك من التسوس والحرص على نظافتها وغسيلها مرتين كل يومً بالفرشاة و معجون الأسنان هيساعده يندمج ويلعب مع أصحابه وجيرانه بثقه و بدون الشعور بإحراج | confidence | friends | positive |
| 1. Brushing your children’s teeth regularly twice a day using a toothbrush and toothpaste means healthier teeth, and this will help them feel and look better in front of their friends and neighbours. | غسيل أسنان طفلك بانتظام مرتين كل يوم بالفرشاة ومعجون الأسنان معناه أسنان صحية أكتر. و ده هيساعده يكون عنده مظهر وشعور أفضل أمام أصحابه وجيرانه | aesthetics | friends | positive |
| 1. Neglecting cleaning your children’s teeth makes it prone to decay and early tooth loss. They might not be able to pronounce words properly and this might affect their confidence in front of their friends and neighbours. That's why it’s important to help your children brush their teeth twice every day using a toothbrush and toothpaste. | عدم غسيلك لأسنان طفلك بيخليها تسوس وتقع بدري وممكن يخليه ما يقدرش ينطق الكلام بشكل سليم ومايكونش عنده ثقة في نفسه قدام أصحابه وجيرانه. عشان كدة ساعديه يغسل أسنانه مرتين كل يوم بالفرشاة ومعجون الأسنان | talk | friends | negative |
| 1. Problems with your children’s teeth can make them embarrassed to play with their friends, making their friends leave them. That’s why it is important to help your children brush their teeth twice a day using a toothbrush and toothpaste. | المشاكل في أسنان طفلك ممكن تخليه يتكسف يلعب مع أصحابه ويخليهم يسيبوه، عشان كده مهم أنك تساعديه ينظف أسنانه مرتين كل يوم بالفرشاة ومعجون أسنان | confidence | friends | negative |
| 1. It is important that the cost of dental treatment to be as low as possible for your family. So, encourage your children to brush their teeth twice a day using a toothbrush and toothpaste. | مهم لأسرتك ان ثمن علاج الأسنان يكون أقل حاجة ممكنة، عشان كده شجعي طفلك يغسل أسنانه كل يوم مرتين بالفرشاة ومعجون الأسنان | money | family | positive |
| 1. The opinion of your family matters. Brushing your children’s teeth twice a day using a toothbrush and toothpaste will help them have a more beautiful smile in front of their family. | رأي عائلتك وأسرتك مهم. غسيل أسنان طفلك مرتين كل يوم بالفرشاة ومعجون الأسنان هيساعده يكون عنده ابتسامة أجمل أمام الأسرة | aesthetics | family | positive |
| 1. Your family pays attention to the appearance and cleanliness of your children’s teeth. Help your children look better by brushing their teeth twice a day using a toothbrush and toothpaste. | عائلتك بينتبهوا لمظهر أسنان طفلك ونظافتها. خلي مظهره أفضل بغسيلها كل يوم مرتين بالفرشاة ومعجون الأسنان | aesthetics | family | positive |
| 1. Your children’s blending with the rest of the children in your family is important. Healthy and clean teeth will help your children blend with the family and feel confident in themselves. That's why help them brush their teeth twice a day using a toothbrush and toothpaste. | اندماج طفلك مع باقي أطفال الأسرة مهم. الأسنان السليمة والنظيفة هتساعد طفلك يندمج مع العائلة ويشعر بثقة في نفسه. عشان كده اغسلي له أسنانه مرتين كل يوم بالفرشاة ومعجون الأسنان | confidence | family | negative |
| 1. When you protect your children's teeth from decay and take care of its cleanliness and brush it twice a day using a toothbrush and toothpaste, they will blend and play with the rest of the children in the family with confidence and without feeling embarrassed. | أما تحافظي على أسنان طفلك من التسوس وتاخدي بالك من نظافتها وغسيلها مرتين كل يوم بالفرشاة ومعجون الأسنان هيندمج ويلعب مع باقي الأطفال في العائلة بثقة وبدون الشعور بإحراج | confidence | family | positive |
| 1. How your children look in front of the family is important. Brushing your children's teeth regularly twice a day using a toothbrush and toothpaste means healthier teeth. This will help them have a better appearance in front of their family. | مظهر طفلك أمام العائلة مهم. غسيل أسنان أطفالك بانتظام مرتين كل يوم بالفرشاة ومعجون الأسنان معناه أسنان صحية أكتر. و ده هيساعده يكون عنده مظهر أفضل أمام العائلة | esthetics | family | positive |
| 1. Neglecting brushing the teeth makes it prone to decay, falling, affects correct pronunciation of words, and affects the children’s self-confidence in front of their family. That's why help your children brush their teeth twice a day using a toothbrush and toothpaste to protect their teeth. | اهمال غسيل الأسنان بيخليها تسوس وتقع ويؤثر على نطق الكلام بشكل سليم ويضعف الثقة بالنفس أمام العائلة. عشان كده ساعدي طفلك إنه يغسل أسنانه مرتين كل يوم بالفرشاة ومعجون الأسنان لحمايتها | talk | family | negative |
| 1. Your children’s tooth loss at a young age due to dental decay and lack of hygiene makes them feel embarrassed and different than the other children. | فقدان طفلك لأسنانه في سن صغير بسبب التسوس وعدم النظافة بيخليه يشعر بالاختلاف عن باقي الأطفال ويكون مكسوف | confidence | general | negative |
| 1. Participating in events and gatherings with your family is important. When your children’s teeth fall out due to decay, it makes them unable to participate in family gatherings and invitations. Help them maintain their healthy teeth by brushing it using a toothbrush and toothpaste. | مشاركة الأقارب في المناسبات والتجمعات مهمة. أما طفلك أسنانه بتقع بسبب التسوس، ده بيخليه مش قادر يشارك في التجمعات والعزومات العائلية. ساعديه يحافظ على أسنانه بغسيلها بالفرشاة والمعجون | confidence | family | negative |
| 1. Healthy and clean teeth makes a person look good. Help your children brush their teeth using a toothbrush and toothpaste. | الأسنان السليمة والنظيفة بتخلي الانسان يكون شكله كويس. ساعدي طفلك ينظف أسنانه بالفرشاة والمعجون | esthetics | general | positive |
| 1. Have you noticed that famous people always have clean and sound teeth? That's why help your children brush their teeth twice every day using a toothbrush and toothpaste. | لاحظتي إن النجوم المشهورين دائماً بتكون أسنانهم نظيفة وسليمة؟ عشان كده ساعدي طفلك إنه يغسل أسنانه مرتين كل يوم بالفرشاة ومعجون الأسنان | esthetics | general | positive |
| 1. Did you notice that in toothpaste advertisements, the teeth are always white and healthy? That's why help your children brush their teeth twice every day using a toothbrush and toothpaste. | لاحظتي إن في إعلانات معجون الأسنان دائماً الأسنان بتكون بيضاء وسليمة؟ عشان كده ساعدي طفلك إنه يغسل أسنانه مرتين كل يوم بالفرشاة ومعجون الأسنان | esthetics | general | positive |
| 1. Healthy teeth will help your children look and feel better about themselves. That's why help them brush their teeth twice every day using a toothbrush and toothpaste. | الأسنان الصحية هتساعد طفلك يكون مظهره و شعوره تجاه نفسه أفضل. عشان كده ساعديه إنه يغسل أسنانه مرتين كل يوم بالفرشاة ومعجون الأسنان. | esthetics | general | positive |
| 1. Brush your children’s teeth using a toothbrush and toothpaste to protect it from decay. Tooth decay can make their mouth smell bad and keep their friends away from him. | اغسلي أسنان طفلك بالفرشاة والمعجون عشان تحميها من التسويس اللي ممكن يخلي ريحة فمه وحشة وتخلي أصحابه يبعدوا عنه | confidence | friends | negative |
| 1. If your children’s teeth become decayed, it may cause a lisp in their speech and affect their self-confidence. Brush their teeth using a toothbrush and toothpaste to protect them. | لو طفلك أسنانه سوست، ممكن تسبب له لدغة في الكلام وتضعف ثقته في نفسه. اغسلي أسنانه بالفرشاة والمعجون عشان تحميها. | talk | general | negative |
| 1. Instead of spending your money on treating your child's decayed teeth, brush their teeth using a toothbrush and toothpaste, and protect it and instead spend your money on something that makes them feel good amongst people. | بدل ما تحتاجي تصرفي فلوسك على علاج أسنان طفلك بسبب التسويس، اغسليها بالفرشاة والمعجون واحميها واصرفي فلوسك على حاجة تخليه يكون كويس وسط الناس. | money | general | negative |
| 1. Brush your child's teeth using a toothbrush and toothpaste, to protect them from decay, and show off your child's teeth in front of people. | اغسلي أسنان طفلك بالفرشاة والمعجون واحميها من التسويس واتباهي بأسنان طفلك وسط الناس. | confidence | general | negative |

**Appendix 3**

**Video links**

**Introduction Video:** [**https://youtu.be/MVkp_zRS5Wc**](https://youtu.be/MVkp_zRS5Wc)

**Video 1:** [**https://youtu.be/9_TKU7D8_X0**](https://youtu.be/9_TKU7D8_X0)

**Video 2:** [**https://youtu.be/SMOWjYlTABs**](https://youtu.be/SMOWjYlTABs)

**Video 3:** [**https://youtu.be/R-3mg6oxLtA**](https://youtu.be/R-3mg6oxLtA)

**Video 4:** [**https://youtu.be/KJGkzYHybwQ**](https://youtu.be/KJGkzYHybwQ)

**Video 5:** [**https://youtu.be/qMRjjbx-07Q**](https://youtu.be/qMRjjbx-07Q)

**Video 6:** [**https://youtu.be/d8hX7ljcl3Y**](https://youtu.be/d8hX7ljcl3Y)

**Video 7:** [**https://youtu.be/7bKutS2ptx4**](https://youtu.be/7bKutS2ptx4)

**Video 8:** [**https://youtu.be/bgFq-AuDWYs**](https://youtu.be/bgFq-AuDWYs)

**Video 9:** [**https://youtu.be/J2IDtbsctuQ**](https://youtu.be/J2IDtbsctuQ)

**Video 10:** [**https://youtu.be/bIuGAxV2FvA**](https://youtu.be/bIuGAxV2FvA)

**Video 11:** [**https://youtu.be/t_02odbFbn0**](https://youtu.be/t_02odbFbn0)

**Video 12:** [**https://youtu.be/_FWM-mEI_Ls**](https://youtu.be/_FWM-mEI_Ls)

**Video 13:** [**https://youtu.be/58LVQKdfcvk**](https://youtu.be/58LVQKdfcvk)

**Video 14:** [**https://youtu.be/5-6pxZVUobQ**](https://youtu.be/5-6pxZVUobQ)

**Video 15:** [**https://youtu.be/8rI5SMv024o**](https://youtu.be/8rI5SMv024o)

**Video 16:** [**https://youtu.be/6yYQWfIvEBw**](https://youtu.be/6yYQWfIvEBw)

**Video 17:** [**https://youtu.be/ZgbBkGd4kys**](https://youtu.be/ZgbBkGd4kys)

**Video 18:** [**https://youtu.be/JOOYY1IZJ8s**](https://youtu.be/JOOYY1IZJ8s)

**Video 19:** [**https://youtu.be/pQ38nOwWPlw**](https://youtu.be/pQ38nOwWPlw)

**Video 20: https://youtu.be/AzX_JqzoCT8**

**Video 21: https://youtu.be/tDHrVaBU0sY**

**Video 22: https://youtu.be/T6WszRrjwt8**

**Video 23: https://youtu.be/YSpI7wB5Utw**

**Video 24: https://youtu.be/304F-yaZc_I**

**Appendix 4**

**Interview Open Ended Qs**

**Domain:**

1. **Affective attitude: “how an individual feels about taking part in the intervention”**

*ايه كان شعورك حول المشاركة في الدراسة؟*

- How did you feel about taking part in the interventions?

1. **Burden: “the perceived amount of effort that is required to participate in the intervention”, including burdens upon time, cognitive effort, or expense**

شايفه نفسك حتحتاجي تبذلي مجهود اد ايه عشان تستخدمي الحاجات اللي في الدراسة، سواء كان وقت ولا تفكير ولا تكلفة مادية؟

- How do you see the amount of effort required to participate in the intervention (including time, thinking, and expenses)?

1. **Ethicality: “the extent to which the intervention is a good fit with an individual's value system”**

*“اد ايه الحاجات اللي في الدراسة مناسبة لقيمك والطريقة اللي بتعيشي بها حياتك؟ شايفه*

- To what extent were the interventions in line with your beliefs/ value system?

1. **Opportunity costs: “the extent to which benefits, profits, or values must be given up engaging in an intervention”**

“إلى أي مدى لازم تتخلى عن حاجات مهمة لكي زي فوائد او مكاسب أو قيم للمشاركة في الدراسة

- To what extent do you feel you have to give up benefits, profits or values to engage in the study?

1. **Perceived effectiveness:
   “extent to which the intervention is perceived to OR perceived to be likely to achieve its purpose”**

“اد ايه شايفه ان الحاجات اللي في الدراسة ممكن تحقق الغرض منها في تحفيز الآمهات ان هم يغسلوا سنان أولادهم ؟"

- To what extent do you believe the things in the study can be effective in motivating parents to brush their children’s teeth and why?

1. **“The participant's confidence that they can perform the behavior(s) required to participate in the intervention”**

ممكن تكلميني عن احساسك بمدى ثقتك في قدرتك على التعامل مع أو استخدام الحاجات اللي في الدراسة؟

- To what extent are you confident that you can deal with or use the intervention components in the study?

1. **Intervention coherence: “the extent to which the participant understands the intervention, and how the intervention works”**

الى أي مدى حسيتي انك فاهمة العناصر المستخدمة في الدراسة والطريقة اللي بيشتغلوا بها؟

- To what extent were you able to understand the activities in the study and how they work?

**Appendix 5**

**Acceptability of Proposed Intervention Components**

**Section 1: Demographics**

**1. Mother ID:**
**2. Mother name:**
**3. Mother age (In years):**

▪19-24

▪25-34

▪35-44

▪45-55

**4. What level of education have you completed? (Mother)**

▪ No formal schooling/ less than primary school
▪ Primary school completed
▪ High school completed
▪ College/university completed
▪ Postgraduate degree

**5. Occupation: (Mother)**

▪ Does not work / Housewife
▪ Unskilled manual worker (porters - day laborers - messengers)
▪ Skilled manual worker (owners of crafts and industrial industries)

▪ Traders and businessmen
▪ Government employees and administrators
▪ Professionals (doctors - university professors - lawyers - officers)

**Section 2: Perceived and Experienced Acceptability
Please indicate to which extent you agree with the following statements**

**Storytelling videos**

1. I enjoyed watching the video

- Strongly agree
- Agree
- Undecided
- Disagree
- Strongly disagree

1. I needed to exert effort to watch the videos (whether time, thinking, or cost)

- Strongly agree
- Agree
- Undecided
- Disagree
- Strongly disagree

1. It was easy to understand and follow the content of the video

- Strongly agree
- Agree
- Undecided
- Disagree
- Strongly disagree

1. The video is inappropriate to my values ​​and the way I live my life

- Strongly agree
- Agree
- Undecided
- Disagree
- Strongly disagree

1. I had to give up important things for example: benefits, gains, or values to be able to watch the video

- Strongly agree
- Agree
- Undecided
- Disagree
- Strongly disagree

1. The videos will be effective in motivating other mothers to brush their children’s teeth

- Strongly agree
- Agree
- Undecided
- Disagree
- Strongly disagree

**Oral health Promotion Messages**

1. I enjoyed reading the messages

- Strongly agree
- Agree
- Undecided
- Disagree
- Strongly disagree

1. I needed to exert effort to read the messages (whether time, thinking, or cost)

- Strongly agree
- Agree
- Undecided
- Disagree
- Strongly disagree

1. It was easy to understand and follow the content of the messages

- Strongly agree
- Agree
- Undecided
- Disagree
- Strongly disagree

1. The messages were inappropriate to my values ​​and the way I live my life

- Strongly agree
- Agree
- Undecided
- Disagree
- Strongly disagree

1. I had to give up important things for example: benefits, gains, or values to be able to read the messages

- Strongly agree
- Agree
- Undecided
- Disagree
- Strongly disagree

1. The messages will be effective in motivating other mothers to brush their children’s teeth

- Strongly agree
- Agree
- Undecided
- Disagree
- Strongly disagree

**Motivational Interview Sessions**

1. I enjoyed receiving the motivational interview session

- Strongly agree
- Agree
- Undecided
- Disagree
- Strongly disagree

1. I needed to exert effort to receive the motivational interview session (whether time, thinking, or cost)

- Strongly agree
- Agree
- Undecided
- Disagree
- Strongly disagree

1. It was easy to understand and follow the content of the motivational interview session

- Strongly agree
- Agree
- Undecided
- Disagree
- Strongly disagree

1. The motivational interview session was inappropriate to my values ​​and the way I live my life

- Strongly agree
- Agree
- Undecided
- Disagree
- Strongly disagree

1. I had to give up important things for example: benefits, gains, or values to be able to receive the motivational interview session

- Strongly agree
- Agree
- Undecided
- Disagree
- Strongly disagree

1. The motivational interview session will be effective in motivating other mothers to brush their children’s teeth

- Strongly agree
- Agree
- Undecided
- Disagree
- Strongly disagree

**Section 3: Preferred frequency and timing of receiving the components**

**I prefer to receive the messages/ videos:**

- 2 times/ week
- 1 time/ week
- 3 times/ month
- 2 times/ month
- 1 times/ month
- Less than that (please define): .........

**I prefer if the messages/ videos are sent:**

- After 8am to 2pm
- After 2pm to 8pm
- After 8pm to 2am
- After 2am to 8am

**Section 4: Specifying an Optimization objective**

**What is the maximum total time are you willing to spend receiving education on** **how to brush your child’s teeth?**

- 2.5-15 min
- >15-30 min
- >30-45 min
- 45-51 min
